# Rats remind us what actually counts in episodic memory research

**DOI:** 10.3389/fpsyg.2015.00075

**Published:** 2015-02-04

**Authors:** Benjamin M. Basile

**Affiliations:** Section on the Neurobiology of Learning and Memory, Laboratory of Neuropsychology, National Institute of Mental Health, National Institutes of HealthBethesda, MD, USA

**Keywords:** episodic, semantic, source, context, rat

Think back to lunch yesterday. You likely remember what you ate, where you sat, who you were with, what you talked about, and myriad other details combined in a cohesive memory. Now think back to lunch the day before yesterday. Though many of the details are probably the same, you likely do not have trouble discriminating between the two memories. This example highlights that episodic memories, or memories for specific experiences, contain many details bound together in cohesive representations (Tulving, 1972; Ranganath, 2010). Contrast episodic with semantic memories, or memories for facts. You may know where the cafeteria is and that sandwiches are food, but those memories are unbound to specific events.

Tulving originally defined episodic memory as encoding dated episodes and their spatial relations (1972), or “what,” “when,” and “where.” These criteria were objective and testable. As the study of episodic memory matured, the goal posts for identifying it were repeatedly moved away from objective criteria and toward subjective ones (reviewed in Tulving, 2002). The modern definition rests heavily on concepts like autonoetic, or self-knowing, consciousness, which gives episodic memory a phenomenal “flavor of pastness” (Tulving, 1984) and a personal “warmth and intimacy” (Tulving, 2001). Consequently, researchers of human memory often rely on participants' verbal reports of whether their memory is subjectively episodic, such as in the classic remember/know paradigm (e.g., Rajaram, 1993; Gardiner, 2001). The result is that we often define episodic memory in the way Supreme Court Justice Potter Stewart famously defined obscenity: “I know it when I see it” Stewart (1964).

Subjective reports limit the questions researchers can answer. Imagine explaining the subjective feel of episodic remembering to a person who has likely never had an episodic memory due to early brain damage (Baddeley et al., 2001). Or testing whether a bee remembers a flower's location with an intimate flavor of pastness. This clash between the subjective criteria of episodic memory and the need for objective measures, especially within comparative psychology, has produced much theory-driven running in circles about what “counts” as episodic memory, but with little progress (e.g., Clayton et al., 2003; Tulving, 2005; Suddendorf and Corballis, 2007).

Fortunately, researchers of comparative psychology have made substantial empirical progress by ignoring, for now, the subjective criteria for episodic memory and instead asking how different species remember past events (Templer and Hampton, 2013b). A recent report from Crystal and Smith (2014) highlights the strength of this approach. Episodic memories are characteristically cohesive; consider the opening example. To test the degree to which rats' memories were similarly bound into cohesive episodes, Crystal and Smith tested rats' memories for foraging episodes that shared many individual features. In each episode, rats encountered several pieces of food on an eight-arm maze. If they found a piece of chocolate themselves, another piece would be in the same location later. But if they learned the chocolate's location by being placed there by the experimenter, or if they found rat chow, that location would be empty later. They engaged in two foraging episodes on identical mazes in two different rooms, which could be differentiated by global visual cues. Thus, the two foraging episodes shared many overlapping features. To earn as much chocolate as possible, rats had to remember not only which food they found, where they found it, how they learned that information, and in which room they foraged, but also bind those features together into a cohesive memory that was distinguishable from another memory with similar features.

The rats successfully remembered the foraging episodes, revisiting the replenishing chocolate more often than the non-replenishing chocolate. Importantly, they did not confuse the two similar foraging episodes. They remembered two episodes as accurately as they remembered one. They remembered episodes that shared many features as accurately as episodes that shared few features. They could even distinguish the two episodes following a week's delay, demonstrating that their memory was long-lasting, like many human episodic memories. The absence of confusion suggests the rats did not remember each foraging event as a collection of isolated features, but rather as a cohesive whole.

This study is one of a growing number within comparative psychology, many by Crystal and colleagues, that highlight how empirically productive the field can be when it focuses on testable criteria. Clearly, there is value to knowing whether nonhumans and humans remember past events similarly. We want to know how similar we are to other species, know how cognitive processes evolved, and identify behaviorally-valid animal models for biomedical advancement. But we cannot test the subjective feeling of pastness in nonhumans. We must identify similarities and differences by focusing on those characteristics of episodic memory that are objectively testable. We know more than ever about the degree to which other species remember multiple features of events (Clayton and Dickinson, 1998; Hampton et al., 2005; Babb and Crystal, 2006), the source of information (Crystal et al., 2013), incidental information (Zentall et al., 2001; Zhou et al., 2012), the temporal order of events (Fortin et al., 2002; Templer and Hampton, 2013a), recollectively (Menzel, 1999; Fortin et al., 2004; Basile and Hampton, 2011), explicitly (Sutton and Shettleworth, 2008; Basile et al., 2015), and over long periods of time (Crystal and Babb, 2008; Martin-Ordas et al., 2013). This list is not exhaustive, but it shows that the field has amassed an impressive knowledge of how different species remember past events, revealing both similarities and differences.

Because we have made little progress in the theoretical debate about what “counts” as episodic memory, and great progress in the empirical study of how different species remember events, the field might benefit from a more explicit rejection of the very debate itself. It is easy to get caught up in the debate about what “counts” as episodic memory, and to see each new piece of evidence as weighing on one side or the other. The recent report from Crystal and Smith should remind us that we make the most progress when we ignore intractable theoretical debates and continue gathering data.

## Conflict of interest statement

The author declares that the research was conducted in the absence of any commercial or financial relationships that could be construed as a potential conflict of interest.
